# *ω*-3 Polyunsaturated Fatty Acids Facilitate the Repair of Peripheral Nerve Defects with Chemically Extracted Acellular Allograft in Rats

**DOI:** 10.1155/2021/2504276

**Published:** 2021-01-26

**Authors:** Jun Zuo, Yuan Wu, Renkun Xiang, Zhiping Dai, Yi Zhu

**Affiliations:** ^1^Second Affiliated Hospital of Nan Hua University, No. 35 Jiefang Road, Zhengxiang District, Hengyang City, Hunan Province 421001, China; ^2^Hengyang Third People's Hospital, No. 111 HongShuang Road, Hengyang County, Hengyang City, Hunan Province 421001, China

## Abstract

Acellular allograft (ACA) improves the repair and reconstruction of long peripheral nerve defects. *ω*-3 Polyunsaturated fatty acids (PUFAs) carry a neuroprotective potential, and their effects on ACA bridging were elucidated. Thirty rats with long gap sciatic nerve defects (15 mm long) were randomly divided into three groups (*n* = 10): ACA, ACA + PUFAs, and autograft (AU). Limb condition, wet weight of tibialis anterior muscle (TAM), nerve electrophysiology, S-100, horseradish peroxidase (HRP), and percentage of splenic CD4+ and CD8 + T-lymphocytes were evaluated for 12 weeks after the operation. Rats in the AU and ACA + PUFA groups showed superior condition in affected limbs compared to the ACA group. At 12 wk after surgery, the wet weight of TAM in the ACA + PUFA group was higher than that in the ACA group (0.4519 ± 0.1185 vs. 0.3049 ± 0.1272; *P* < 0.01) but lower than that in the AU group (0.4519 ± 0.1185, 0.5628 ± 0.0092; *P* < 0.05). In all the three groups, sole irritation elicited withdrawal reflex, and S-100 staining was detected in plantar skin. Moreover, horseradish peroxidase staining was overt in both the ventral horn and dorsal root ganglion of the spinal cord. Nerve conduction velocity (m/s), amplitude of action potential (mV), or somatosensory evoked potentials in ACA + PUFAs (28.81 ± 1.04, 2.20 ± 0.27, 6.98 ± 0.29) were significantly different from that in the AU (35.71 ± 1.28, 1.81 ± 0.19, 8.15 ± 0.52; *P* < 0.05) and ACA (20.03 ± 1.94, 2.95 ± 0.36, 5.22 ± 0.53; *P* < 0.01) groups. The percentages of splenic CD4+ and CD8+ cells were similar among the three groups. Omega-3 PUFAs improve the bridging effect of ACA on long gap peripheral nerve defects by promoting neuroprotection without arousing an immune response.

## 1. Introduction

Peripheral nerve injuries are common issues in clinical practice, which are ascribed to various causes including sharp or blunt forces, stretching, and crushing. This type of nerve damage comes with functional disorders, featured by declining skeletal muscle strength and hypoesthesia in the innervated areas [1]. Repair and reconstruction of nerve defects are critical neurosurgical issues. Nerve autograft (AU) is regarded as the most efficient standard approach for bridging peripheral nerve defects. However, it simultaneously causes drawbacks including secondary trauma, motor and sensory forfeit in donor sites, scarring, and postoperative neuromatous pain [2]. Moreover, the shortage of donor sources makes it problematic to find proper matching graft for the damaged nerve, especially for long, multiple, or large nerve defects. Mesenchymal stem cells (MSCs) [3–5] and nerve conduit [6] are alternative measures for the repair and reconstruction of peripheral nerve defects. Among them, allogenic nerves, when deprived of immunogenic components such as Schwann cells, contain only reticular columnar structure, the length of which can be adjusted without trouble to accommodate the requirements of the recipient [7]. Therefore, in addition to AU, acellular allograft (ACA) is able to bridge peripheral nerve gaps, especially the absence of the large nerve. Recently, the ACA has been applied in clinical practice, exhibiting notable efficacy. Even so, this approach does not keep pace with AU in therapeutic outcomes; thus, measures with the aim of advancing the bridging capacity of ACA should be pursued.

Dietary polyunsaturated fatty acids (*PUFA*s), especially *ω-*3 PUFAs, have shown prominent roles in antioxidation and immunoregulation [8]. Moreover, the neuroprotective performances of Omega-3 PUFAs have been demonstrated in both animal and human studies [9]. Dietary supplementation with *ω*-3 PUFAs attenuates neuronal damage in various situations. Docosahexaenoic acid (DHA) and eicosapentaenoic acid (EPA) accelerate rebuilding of the structural and function neurovascular unit (NVU) in neonatal rats suffering from cerebral ischemic-hypoxic injury [10]. DHA promotes axon growth by enhancing the expression of neuroprotective genes such as B cell lymphoma-extralarge, B cell lymphoma 2 (Bcl-2), and Bcl-2-related protein A1 but reducing that of proapoptotic genes such as Bcl-2-associated X protein and B cell 2-interacting killer [11, 12]. Thus, it has been hypothesized that the combination of ACA and Omega-3 PUFAs might act synergistically to bridge large periphery nerve defects. This hypothesis was tested in animal models.

In this work, a long gap sciatic nerve defect was established in rats, on which ACA bridging was conducted. Then, the rats were given Omega-3 PUFAs in their diet for 12 weeks, during which time the functional recovery of the nerve, local inflammation, and systemic immune response was assessed.

## 2. Materials and Methods

### 2.1. Animal Grouping and Feeding

Thirty Sprague-Dawley (SD) rats with long gap sciatic nerve defects (15 mm long) were randomly divided into three groups (*n* = 10): ACA, ACA + PUFAs, and AU. Before surgery, all rats had the same housing and feeding conditions. After the bridging operation, rats in the ACA + PUFAs group were fed a diet enriched with Omega-3 PUFAs (1 g fish oil/20 g regular chow, 00001141; Fresenius Kabi Austria GmbH company). In the other two groups, the rats were given regular chow. All procedures were conducted based on the guidelines raised by the Ethics Committee of the International Association for the Study of pain [13]. The Research Council of our university approved all of the experimental procedures (SYXK 2015-0001).

### 2.2. Sciatic Nerve Defect Establishment and Bridging

SD rats were anesthetized by sodium pentobarbital administered intraperitoneally (30 mg/kg). The skin in the right buttock and thigh was prepared and sterilized, after which a 2–4 cm curved incision was made. The gluteal muscles were bluntly dissected to expose the sciatic nerve. Then, 15 mm of unbranched sciatic nerves was resected to establish the sciatic nerve defects. The bridging operations were conducted under magnifying lenses (ASOM-4; Corder Co., Chengdu, China). The nerve stumps were connected together with 10-0 atraumatic sutures, and then, the muscle and skin were closed. After the operation, the rats were housed (1–2 animals/cage) and given 2.0 g Amoxicillin granules for 3 days. The general conditions of the rats including consciousness, eating, wound, activity, muscle atrophy, foot ulcer, and toes of the affected limbs were observed and recorded.

### 2.3. Acellular Nerve Allograft Preparation

Five SD rats were anesthetized as above. The skin in the bilateral thigh was sterilized. The posterolateral sciatic nerve was exposed and isolated at the unbranching regions. Then, the nerves were cut into 15 mm of sections, which then underwent modified acellular treatment [14]. Briefly, the nerve segments were incubated in distilled water at 25°C for 7 h with continuous shaking by 80 times/min and then in decyldimethyl (3-sulfopropyl) ammonium hydroxide inner salt (SB-10) for 15 h which was followed by shaking in sterilized phosphate-buffered saline (PBS) for 15 min. Next, the nerve was immersed in hexadecyldimethyl (3-sulfopropyl) ammonium hydroxide inner salt (SB-16, *v*/*v*, 0.006%) and Triton X-200 (*v*/*v*, 0.14%) for 24 h, followed by shaking in sterilized PBS three times for 15 min each. Then, the nerve segments were placed in sterilized PBS at 4°C. Normally, the allografts were prepared for 69.5 h before being used, during which time they were preserved in sterilized PBS at 4°C.

### 2.4. Electroneurophysiological Test

Twelve weeks after operation, the bridged sciatic nerves were isolated by opening the original incisions. The corresponding sciatic nerves in the healthy side were also isolated. Two steel wire electrodes were placed 10 mm above and below the bridging segment, acting as the stimulator (S) and receiver (R), respectively. Nerve conduction velocity (NCV, m/s), amplitude of action potential (NAP, mV), and somatosensory evoked potential (SEP, mV) were recorded. The NCV was calculated as length (S-R)/time of transduction. The consequences of affected and healthy sides were compared.

### 2.5. Tibialis Anterior Muscle Wet Weight Recovery Rate Measure

The tibialis anterior muscle (TAM) of the affected and controlateral limbs was harvested, and the wet weights were assessed using an analytical balance (EX324; OHAUS, Parsippany, NJ, USA) after removing the blood with filter paper. The TAM recovery rate after bridging was calculated as the wet weight of the affected limbs divided by that of the contralateral limbs.

### 2.6. Horseradish Peroxidase Retrograde Labeling

Twelve weeks after the operation, the SD rats were anesthetized by sodium pentobarbital administered intraperitoneally (30 mg/kg). A 3–4 cm curved incision was made in the right limbs from buttock to thigh to expose the sciatic nerve. At the far end of the graft, 4 *μ*L of 30% horseradish peroxidase (HRP) (RZ3.2; Roche, Basel, Switzerland) was injected, and the needles were retained *in situ* for 10 min. Then, the nerve was severed 0.5 cm below the anastomosis, and the HRP crystal was applied to the near broken end. Subsequently, the nerve graft was immersed in 10% HRP and placed in a plastic tube sealed with solid Vaseline. The incision was closed, and the rats were raised for another 72 h. Next, the chest of rats was opened for left ventricular intubation under anesthesia with pentobarbital infusion (30 mg/kg). The blood was flushed with PSS containing heparin, and then, paraformaldehyde (20 g/L) and glutaraldehyde (12.5 g/L) in PBS (0.01 mol/L, 4°C, pH 7.4) were perfused for 2 h to fix the tissues. Ganglia and corresponding spinal cords in L4, 5, and 6 were isolated and fixed *in vitro* for 6 h, after which the tissues were placed overnight at 4°C in 0.01 mol/L of PBS containing 300 g/L sucrose solution. Next, 30 *μ*m of successive frozen sections was prepared and stained with DAB, and HRP average absorbance was measured (HPIAS-1000; Buehler, Lake Bluff, IL, USA).

### 2.7. Immunohistochemistry Staining of S-100 in Plantar Skin

The slides were dried at 68°C for 20 min and then underwent conventional dewaxing, which next were incubated in 3% H_2_O_2_ at 37°C for 10 min followed by washing three times in PBS for 5 min each. Next, the sections were placed in citric acid buffer (pH 6.0) for 15–20 min at 95–100°C followed by cooling at room temperature to retrieve the antigens. Then, the sections were incubated in normal goat serum for 10 min at 37°C, followed by incubation with primary antibody overnight at 4°C. After being washed with PBS, the slides were incubated with biotinylated secondary antibody for 10–30 min at 37°C. Then, an additional wash with PBS was performed following by incubating the sections with HRP reaction solution at 37°C for 10–30 min. Finally, another PBS wash and DAB staining were conducted before the S-100 expression was examined under a microscope (CX23; OLYMPUS, Tokyo, Japan).

### 2.8. Flow Cytometry to Assess the Percentage of T Cells

The spleen was minced and ground gently on 400 nylon meshes. After rinsing with PBS, the splenocytes were transferred to a plastic tube for centrifugation at 1500 pm for 10 min. The supernatants were discarded, and the pellets were collected. Then, 3 mL of erythrocyte lysate was added mixed at 4°C for 5 min, followed by centrifugation at 1500 r/min for 10 min. The supernatant was discarded, and the cells were again washed with PBS, followed by a 10 min of centrifugation at 1500 rpm. After two washes and another centrifugation, the supernatant was removed, and spleen mononuclear cells were collected. Next, the cells were suspended in RPMI 1640 and adjusted to a concentration of 5 × 10^6^/mL which were then incubated with phorbol 12-myristate 13-acetate (50 ng/mL), ionomycin (1 *μ*g/mL), and brefeldin A (10 *μ*g/mL) at 37°C with 5% CO_2_ for 5 h, followed by a 10 min centrifugation at 1500 rpm. The supernatant was discarded. Following another wash with PBS, a 15 min of incubation with PerCP/cy5.5 anti-mouse CD3 and FITC anti-mouse CD4 (eBioscience, San Diego, CA, USA) was carried out at 4°C in the dark. After centrifugation, the supernatant was discarded, and the cells were resuspended in 100 *μ*L of permeabilization buffer. The cells were incubated with anti-mouse IL-17 antibody at 4°C for 20 min, followed by centrifugation in 2 mL PBS with 1% BSA at 1500 rpm for 10 min. The supernatant was discarded, and the cell pellet was dispersed in 500 *μ*L of PBS. Then, the cells were stored at 4°C away from light for detection in a flow cytometer (BD FACS Calibur; BD Biosciences, Franklin Lakes, NJ, USA).

### 2.9. Statistical Analysis

Data are expressed as the means ± standard deviations. The Student's *t*-test was used to determine the significance of differences between groups. Statistical analysis was performed by SPSS 13.0 (IBM, Armonk, NY, USA). *P* < 0.05 was considered statistically significant.

## 3. Results

### 3.1. General Information of Rats in the Three Groups

**Figure 1:**
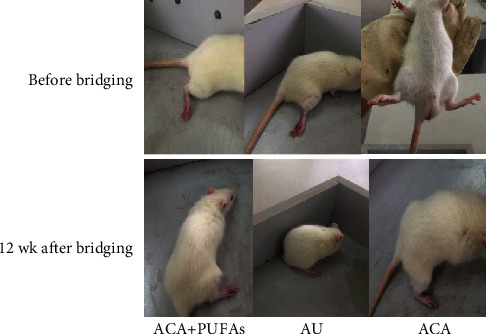
The manifestation of affected toes in the ACA + PUFAs, AU, and ACA groups before and at 12 wk after sciatic nerve defect establishment and bridging. ACA: acellular allograft; PUFAs: polyunsaturated fatty acids; AU: autograft. *n* = 30.

No rat in any of the three groups died during the study. After the operation, all rats showed abnormality in the affected limbs including limited mobility, resistance to touching the ground, and closed and curled toes. After about 1 wk recovery, the incisions were healed, and the affected lower limbs exhibited more or less swelling and festering. At 4 wk after surgery, the swelling gradually subsided. The toes of the rats in both the AU and ACA + PUFA groups showed no atrophy, which however occurred in some rats in the ACA group. Gastrocnemius atrophy appeared in rats of all the three groups. After 12 wk of recovery, the rats in the AU group could separate affected toes, and their limbs were able to touch the ground. By contrast, the rats in the ACA and ACA + PUFA groups presented with slight resumption of atrophied gastrocnemius, and the limbs were unable to touch the ground. The withdrawal reflex triggered by irritating sole skin appeared in rats of the ACA and ACA + PUFA groups but the AU group (Figure 1).

### 3.2. Neuroelectrophysiological Performance of Rats in the Three Groups

To evaluate the functional recovery of nerves, the NCV, NAP, and SEP of neuroelectrophysiological tests were measured. As shown in Table 1, the NCV in ACA + PUFAs was 28.81 ± 1.04, which was higher than that in the ACA group (20.03 ± 1.94, *P* < 0.01) but lower than that in the AU group (35.71 ± 1.28, *P* < 0.05). The amplitude of NAP was 2.20 ± 0.27 in the ACA + PUFA group, which was lower than that in the ACA group (2.95 ± 0.36; *P* < 0.01) but higher than that in the AU group (1.81 ± 0.19; *P* < 0.05). The amplitude of SEP was 6.98 ± 0.29 in the ACA + PUFA group, which was higher than that in the ACA group (5.22 ± 0.53; *P* < 0.01) but lower than that in the AU group (8.15 ± 0.52; *P* < 0.05).

### 3.3. TAM Wet Weight of the Three Groups

**Figure 2:**
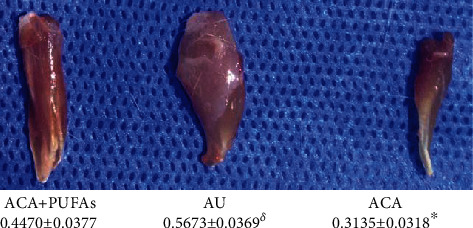
The wet weight recovery of TAM in the ACA + PUFAs, AU, and ACA groups at 12 wk after sciatic nerve defect establishment and bridging. *n* = 30^*δ*^, *P* < 0.05 vs. ACA + PUFAs; ^★^*P* < 0.01 vs. ACA + PUFAs.

The TAM wet weight recovery rate of the affected limbs in the ACA + PUFA group was lower than that in the AU group (0.4470 ± 0.0377 vs. 0.5673 ± 0.0369; *P* < 0.05) but higher than that in the ACA group (0.3135 ± 0.0318; *P* < 0.01) (Figure 2).

### 3.4. HRP Retrograde Labeling in the Three Groups

Varying amounts of HRP-positive cells were detected in both the ventral horn and dorsal root ganglion of the spinal cords in the three groups. However, there was no significant difference between any two groups (Table 2).

### 3.5. S-100 Expression in the Plantar Skin of Rats in the Three Groups

More or less S-100-positive cells were found in the planter skin of rats in all three groups. The absorbances in the rats of the ACA + PUFA, AU, and ACA groups were 0.54 ± 0.04, 0.51 ± 0.04, and 0.49 ± 0.05, respectively. The difference between any two groups did not reach statistical significance (Table 3).

### 3.6. T Cell Proliferation in the Three Groups

**Figure 3:**
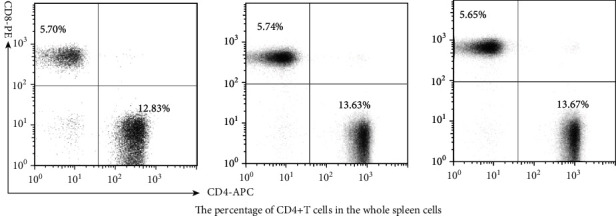
Percentage of CD4+ and CD8+ cell in whole spleen cells in the ACA + PUFAs, AU, and ACA groups at 12 wk after sciatic nerve defect establishment and bridging. *n* = 30.

The percentages of CD4 + T cells in the whole spleen cells were 12.83 ± 0.46, 13.63 ± 0.57, and 13.67 ± 0.46 in the rats of the ACA + PUFA, AU, and ACA groups, respectively. The percentages of CD8+ cells in whole spleen cells were 5.70 ± 0.35, 5.74 ± 0.44, and 5.65 ± 0.46 in the ACA + PUFA, AU, and ACA groups, respectively. There was no significant difference in the abundance of CD4+ or CD8+ T cells between any two groups (Figure 3).

## 4. Discussion

In this study, long gap sciatic nerve defects were established, based on which effects of Omega-3 PUFAs on ACA bridging were delineated. The results showed that the PUFAs significantly improved the functional recovery of nerves in the ACA group but still could not emulate the therapeutic potency of AU. The percentage of CD4+ and CD8 + T cells, which indicated the intensity of the immune response, was similar among the three groups.

Peripheral nerves are close to the surface of body apt to be injured by mechanical stimuli such as cutting, stretching, and compression. The diminished activity of the nerves was manifested by the blunted strength of muscle innervation and hypoesthesia in the regions controlled by the nerves [15]. Peripheral nerve defects cause serious economical and mental loss to patients, so pursuing repair and reconstruction approaches has always been a main neurosurgical subject. Currently, peripheral nerve defects are manipulated by surgery, tissue engineering, gene therapy, and nerve grafting, which include autografting, allografting, and xenografting [16]. AU is recognized as the “golden standard” for nerve bridging, which however is limited by scarcity of resources as well as secondary sensory and motor disorders in donor sites [17]. In recent years, various nerve conduits have stood out in the repair of peripheral nerve injury, especially for the large and segment defects, which has become a crucial alternative strategy for this condition. Among them, ACA has shown the most potentiality [14]. This technique removes most immunogens such as Schwann cells by chemical means and leaves only the reticular nerve structure of nerves, which substantially neutralizes immunologic rejection [2]. In addition, in the ACA approach, the nerve length can readily be adjusted as required, which constitutes an advantage over other synthetic nerve conduits. Even so, ACA is still incomparable to AU with regard to therapeutic outcomes. Therefore, exploring adjuvant measures to enhance ACA in the peripheral nerve defect bridging is a substantial task in this context.

*ω*-3 PUFAs, with DHA and EPA as representative components, clearly improve the integral nutritional status, which also show prominent neuroprotective characteristics. Dietary supplementation with *ω*-3 PUFAs promotes nerve regeneration and NVU reconstruction [18]. Many studies have substantiated that foods enriched in Omega-3 PUFAs alleviate ischemia/hypoxia-induced brain damages, accelerate brain development of infants, and reduce the severity of depression or Alzheimer's disease [19]. The underlying mechanism involves regulating lipid metabolism, modifying gene expression for axon growth, and stabilizing the oxidation/antioxidation balance [20].

In the present work, Omega-3 PUFAs synergistically enhanced the bridging effect of ACA in long gap sciatic nerve defects, which was manifested by the recovery of nerve function and wet weight of TAM. This result suggests the Omega-3 PUFAs could be adopted to repair and reconstruct the large periphery nerve defect. However, the therapeutic potency of ACA with the aid of Omega-3 PUFAs still could not reach the efficiency of AU which was in accordance with previous reports suggesting the Omega-3 PUFAs hold finite value for neural growth [21]. In view of this situation, multiple approaches should be undertaken to compensate for the deficiency of each one for achieving the greatest benefits in managing nerve injury.

S100 stands for a group of calcium-binding proteins with a low molecular weight, among which S100A1 resides in the cytoplasm of neurocytes, skeletal muscle cells, cardiomyocytes, and nephrocytes. Unlike S100A1, S100B is mainly located in neuroglial cells, Schwann cells, and neurons of the central and peripheral nervous systems [22]. So in research on periphery nerve defect repair, S100 abundance in the plantar skin has been regarded as one of the main indicators of functional recovery of sensory nerve endings. On the other hand, the HRP retrograde tracing technique provides morphological evidence for the regeneration of nerve, recovery of axoplasmic flow, and electroneurophysiology [23]. In the present work, both S100- and HPR-positive cells were found in the affected limbs and corresponding spinal segments of rats. However, the difference between any two groups did not reach statistical significance. This might be due to the qualitative but not quantitative nature of the two indicators, which does not change proportionally with the severity of nerve damage.

It is well known that Omega-3 PUFAs mitigate inflammation mediated by T cells [24]. In this work, flow cytometry analysis did not show a difference between groups in the percentage of CD4 + T or CD8 + T cells, which indicates that most of the antigens had been depleted in the ACA preparation of grafts, and there was no extra stimulus in PUFA supplementation to elicit an immune response. Moreover, 12 weeks after the operation, when the original surgical field was exposed, the rats in the ACA + PUFA group exhibited lower adhesion and scarring compared to the ACA group, suggesting that local inflammation induced by bridging maneuvers was reversed by the Omega-3 PUFAs. This performance might be attributed to the modulation of Omega-3 PUFAs on proinflammatory cytokines such as TNF-*α* and IL-13.

## 5. Conclusions

In summary, Omega-3 PUFAs enhanced the bridging efficiency of ACA by promoting nerve regeneration and innervated muscle recovery. Therefore, this lipid nutrient may serve as an adjuvant selection for repairing large periphery nerve defects.

## Figures and Tables

**Table 1:** Neuroelectrophysiological performance of rats in the three groups.

Group	NCV (m/s)	NAP (mV)	SEP (mV)
ACA + PUFAs	28.81 ± 1.04	2.20 ± 0.27	6.98 ± 0.29
AU	35.71 ± 1.28^*δ*^	1.81 ± 0.18	8.15 ± 0.52
ACA	20.03 ± 1.94^★^	2.95 ± 0.36	5.22 ± 0.53

NCV: nerve conduction velocity; NAP: nerve action potential; SEP: somatosensory evoked potential.^*δ*^*P* < 0.05 vs. ACA + PUFAs; ^★^*P* < 0.01 vs. ACA + PUFAs. *n* = 30.

**Table 2:** HRP retrograde labeling abundance in three groups.

Group	*n*	HRP
ACA + PUFAs	10	0.66 ± 0.03
AU	10	0.68 ± 0.04
ACA	10	0.65 ± 0.02

HRP: horseradish peroxidase.

**Table 3:** S-100 expression in plantar skin of rats in the three groups.

Group	*n*	S-100
ACA + PUFAs	10	0.54 ± 0.04
AU	10	0.51 ± 0.04
ACA	10	0.49 ± 0.05

## Data Availability

The datasets used and analysed during the current study are available from the corresponding author on reasonable request.
